# Study protocol for measuring energy needs and nutritional status of adolescent girls in Abia state, Nigeria: The nutrition and adolescence in rural Nigeria (NARN’ia) Study

**DOI:** 10.1371/journal.pone.0333889

**Published:** 2025-10-07

**Authors:** Mayowa T. Adegboyega, Chigozirim A. Amaeze, Chinaza P. Uche, Patricia O. Ukegbu, Herman Pontzer

**Affiliations:** 1 Department of Evolutionary Anthropology, Duke University, Biological Sciences, Campus, Durham, North Carolina, United States of America; 2 Deptartment of Human Nutrition and Dietetics, Michael Okpara University of Agriculture, Umudike, Abia State, Nigeria; Dow University of Health Sciences, PAKISTAN

## Abstract

**Background:**

Adolescents in low- and middle-income countries face significant nutritional challenges, including undernutrition, micronutrient deficiencies, poor dietary habits, and food insecurity. These issues adversely affect their growth, development, and long-term health. Despite their vulnerability, adolescents are frequently excluded from nutritional program planning due to limited data, weak intervention delivery platforms, and underdeveloped policies.

**Methods/design:**

The Nutrition and Adolescence in Rural Nigeria (NARN’ia) study aims to address this gap through a comprehensive assessment of energy requirements in adolescent girls (N = 70; ages 10–19 years) in Nigeria. NARN’ia will combine multiple methods: 24-hour dietary recall using the (INDDEX24 app); physical activity monitoring via accelerometry; anthropometric assessments of body composition, and the doubly labeled water method to measure total daily energy expenditure and body composition.

**Discussion:**

Findings from this study will inform evidence-based nutritional guidelines tailored to adolescent girls in Nigeria. By integrating robust methodologies, NARN’ia will contribute foundational data on adolescent energy needs, supporting future research and policy development in Nigeria and other low- and middle-income countries.

**Trial registration:**

ClinicalTrials.gov NCT06601959

## 1. Introduction

Adolescence is a transitionary developmental phase between childhood and adulthood that is characterized by changes in body composition such as changes in the ratio of fat to fat-free mass, increase in skeletal mass, as well as changes in primary and secondary sexual characteristics [1,2]. Adolescence offers a second opportunity for rapid growth and development, and the final window to eradicate stunting [3]. During this period, humans gain up to 50% of their adult weight, more than 20% of their adult height, and 50% of their adult skeletal mass [4]. These growth and maturational processes lead to elevated daily energy requirements during adolescence and require a corresponding increase in nutritional intake [5], however these requirements are not always met.

A critical element in formulating health and nutrition policies for adolescents is a clear understanding of their energy requirements and dietary intake [6]. Energy requirements refer to the amount of food energy necessary to balance total energy expenditure (TEE), supporting physical activity as well as growth and development [7]; Torun, 2005). In the past, these analyses relied heavily on survey-based estimates of TEE, daily physical activity, and diet [8]. However, in recent decades, the development of accelerometry to objectively measure daily physical activity, and the doubly labeled water (DLW) method to objectively measure TEE during normal daily life have improved assessments of these key variables, and instruments for recording dietary intake have improved [5,9–12].

### 1.1. Dietary assessment

Dietary assessment involves measuring food intake, dietary diversity, and feeding behaviors of individuals, groups, or populations. It includes comparing these factors to recommended dietary guidelines and tracking changes in dietary habits over time [13–15]. Self-reported dietary surveys ask participants to detail all food and drink consumed, including portion sizes, locations, and times of consumption [16]. This information is often collected using dietary assessment software linked to food composition databases, which convert reported foods and beverages into energy and nutrient values [17].

The 24-hour dietary recall is a dietary assessment tool in which participants are asked to recall and describe all foods and beverages consumed within the past 24 hours, usually of the previous day. A trained interviewer typically conducts the recall however, the process can also be self-administered using a mobile application. To enhance the accuracy of the recall, the multiple pass method is used. This involves participants providing information on all their consumptions with increasing details such as the variety of produce, preparation methods, and added ingredients over multiple passes [18]. The 24-hour dietary recall method can also be employed to assess dietary diversity and nutritional quality by determining the number of different food groups consumed.

The accuracy of these methods is contingent on several key factors such as participant compliance, i.e., their willingness and ability to accurately describe their food and beverage consumptions [17]; interviewers’ ability to ask the necessary questions and accurately interpret and record participants’ responses; and the accuracy of the food composition database used to convey the reported intakes into energy and nutrient measurements [17,19,20]. Errors in dietary recall can be mitigated through thorough training of both participants and interviewers, along with the use of a reliable food composition database. This multifaceted approach ensures that the dietary data collected accurately reflects the participants’ consumptions.

### 1.2. Physical activity assessment

Our current understanding of physical activity levels among adolescents remains limited by the precision of assessment methods, and the difficulties inherent in measuring habitual physical activity in this demographic [21,22]. These limitations hinder comprehensive insights into the impact of physical activity on adolescent health and development. Low physical activity during adolescence has been associated with increased risk of obesity, cardiovascular diseases, and diabetes [23–25]. Over 80% of adolescents worldwide fail to meet the current recommendations of at least one hour of daily physical activity [4,26–28].

A prior study conducted in Nigeria utilizing activity diaries kept by participants to measure energy expenditure revealed that energy expenditure was low among adolescents with notably higher levels among boys compared to girls across all ages [2]. However, the accuracy of diaries in capturing the patterns of physical activity remains uncertain, underscoring the need for additional research that more comprehensively assesses both the intensity and duration of physical activity over an extended period.

Activity monitoring using accelerometry has been validated for measuring energy cost in children and adolescents [29]. Triaxial accelerometers detect the magnitude and direction of acceleration across all three planes of motion simultaneously [30]. This method is widely used in large-scale studies to assess and quantify human movement and activity patterns [31]. Despite its global popularity, its application in Nigeria has been limited, with only one study identified that employed this technology [32] and none specifically in the population under investigation here. Therefore, this study has the potential to offer unique insights into energy expenditure research in Nigeria and globally, potentially filling crucial gaps in understanding physical activity patterns and their impact on health outcomes in this demographic.

### 1.3. Body composition

Body composition refers to the proportions of fat, muscle, bone, and other tissues that make up an individual’s body mass (BM). The primary components of body composition are fat mass (FM), which includes all fat tissue in the body, and fat free mass (FFM) including muscle, bone, organs, and water [33]. Maintaining a healthy body composition is important for overall health, physical performance, and disease prevention which are influenced by diet, physical activity levels, genetics, and age [34].

Body composition studies are crucial for informing strategic interventions and policies aimed at meeting nutritional needs. However, data on body composition among adolescents, particularly in rural and low to middle-income countries (LMICs), has relied on techniques like anthropometric measurements, e.g., skinfold thickness (SFT), and total body electrical conductivity [35,36]. Anthropometry is a rapid and cost-effective technique for assessing body composition [37], but it is limited in its ability to accurately assess body composition [38,39]. There remains a critical gap in foundational data for adolescents who are experiencing dynamic changes in nutritional needs and metabolism [5].

The use of isotope dilution to measure total body water (TBW) has become one of the main methods for conducting body composition analysis [36,40]. In a model of body composition that includes FM and FFM, water is a crucial component of FFM [41] and because the hydration of FFM remains relatively constant among healthy individuals [42], FFM can be calculated from TBW measurement with deuterium dilution [43]. Since deuterium is one of the isotopes used in DLW assessments of energy expenditure, deuterium dilution assessment of TBW and FFM is a standard output for DLW analyses.

### 1.4. Energy expenditure

The DLW method is also considered the gold standard for estimating TEE under free-living conditions with minimum interference to daily life [5,36,40,44–48]. This technique quantifies the production rate of carbon dioxide (CO_2_) by measuring the differential elimination rates of deuterium (^2^H) and oxygen-18 (^18^O) from the body’s total water pool. Participants drink a dose of water (1.25 grams per kg body weight) that is enriched to 6% ^2^H_2_-O and 10%H_2_-^18^O. Deuterium (^2^H) is eliminated through water in bodily fluids (e.g., urine, expired water vapor, sweat), whereas oxygen-18 (^18^O) exits through both bodily fluids and carbon dioxide (C-^18^O_2_) [49]. The difference in the elimination rates of these isotopes can therefore be used to calculate the rate of CO_2_ production. Carbon dioxide (CO_2_) is a waste product of energy metabolism, and therefore the rate of CO_2_ production can be used to calculate the rate of energy expenditure.

DLW has demonstrated high accuracy in calculating TEE [50–52]. Despite its efficacy, DLW usage is predominantly observed in high-income countries (HICs) [7, 53] largely due to associated prohibitive costs and limited analytical expertise [54, 55]. Similarly, to other LMICs, the use of DLW in Nigeria is limited [56–58] and this forthcoming study focusing on adolescents will be the first of its kind in the region.

The Nutrition and Adolescence in Rural Nigeria (NARN’ia) study will integrate the DLW method with dietary assessments using the 24-hour INDEXX App, anthropometric evaluations of body composition, and accelerometry for physical activity quantification. Together, these methods will provide evidence-based data essential for designing and implementing nutritional programs tailored to the needs of adolescent girls in the region. Furthermore, this research initiative aims to establish a foundational framework for future studies on energy requirements in Nigeria and other LMICs.

### 1.5. Nutritional challenges in Nigeria

There are approximately 600 million adolescent girls (aged 10–19) worldwide, with nearly 90% residing in developing nations [59]. In Nigeria alone, the number exceeds 25 million, representing about 13% of the total population [60,61]. Adolescents worldwide face numerous nutritional challenges that impact their growth, development, and future health. Undernutrition, micronutrient deficiencies, poor dietary practices, and food insecurity are prevalent in low and middle-income countries (LMICs). Increasingly, these issues coexist with rising obesity rates in LMICs, a phenomenon known as the dual burden of malnutrition [62].

A considerable number of adolescent girls across Africa suffer from chronic malnutrition, severely affecting their health and development. In Nigeria specifically, about 1 in 4 adolescent girls (ages 15–19) are malnourished, and 61% are anemic [63]. Despite these alarming statistics, in Nigeria, similarly to most developing countries, nutrition initiatives have primarily focused on young children and/or adult women, leaving this demographic quite neglected [64]. This neglect is concerning because adolescent girls are particularly vulnerable to malnutrition due to their increased need for nutrients, cultural influences that affect their eating habits, and susceptibility to environmental influences prevalent in impoverished regions [65,66]. Addressing their nutritional needs is a crucial step toward breaking the cycle of intergenerational malnutrition, chronic diseases, and poverty [67]. However, solving these challenges and improving adolescent girl nutrition requires accurate and reliable measures of daily energy needs.

NARN’ia aims to conduct the most comprehensive assessment of energy requirements in adolescent girls in Nigeria to date. This study will integrate dietary assessments using the 24-hour recall method; physical activity levels using accelerometry; anthropometric evaluations of body composition, and the DLW method for assessing both body composition as well as indirect calorimetry. Together, these methods will provide evidence-based data essential for formulating appropriate nutritional guidelines to support healthy growth and development of adolescent girls in Nigeria. Furthermore, this research initiative will establish a foundational framework for future studies on energy requirements in Nigeria and other LMICs.

## 2. Methods and project design

### 2.1. Trial registrations and ethical clearances

The study protocol was approved by the Institutional Review Board of the Duke University Health System (Durham, NC, USA; protocol 00114378). The University of Nigeria Teaching Hospital Health Research Ethics Committee (NHREC/o5/01/2008B-FWA00002458–1RB00002323) provided ethical clearance ensuring that the research plan complies with international ethical standards for human subject research. The study was also approved by the Abia State Secondary Education Board. The ClinicalTrials.gov identifier is NCT06601959.

### 2.2. Study participants

The NARN’ia study is being conducted in Ikwuano Local Government Area of Abia State, located in southeast Nigeria. Ikwuano is a rural region primarily inhabited by crop farmers and traders from the Igbo ethnic group. The recruitment goal for the NARN’ia study is a total of 70 adolescent girls selected from four secondary schools in Ikwuano. The schools were selected in consultation with local education authorities based on geographic spread, accessibility, school size, and willingness to participate. Within each selected school, eligible participants are identified. From this pool, students are randomly selected through balloting without replacement.

Inclusion criteria include female students between the ages of 10 and 19 years who provide verbal assent and have written parental consent. Exclusion criteria include students with physical disabilities, those who are ill during the study period, and students attending boarding schools due to their more restricted living conditions.

Due to logistical constraints particularly the delayed and phased delivery of research materials from Duke University to the study site at MOUAU, the study is being conducted in two phases. This phased approach also allows the local team to manage recruitment and data collection more effectively. Importantly, both phases follow the same recruitment procedures, inclusion and exclusion criteria, and data collection methodology to ensure consistency and comparability of results.

In Phase 1, which took place in early 2024, 40 participants were recruited from four secondary schools. In Phase 2, an additional 20 participants are being recruited beginning in early 2025. In both phases, the same types of data are collected, including anthropometric measurements (height, weight, body composition), assessments of physical activity levels, and 24-hour dietary recalls. There are also no methodological differences between the two phases.

The study team has also considered the potential influence of seasonal factors, such as school holidays and variations in food availability and physical activity patterns, particularly between the dry and rainy seasons in Nigeria. While both phases are scheduled to occur during the school term to ensure access to participants and minimize variability due to holidays, possible seasonal differences in food consumption and activity patterns will be documented and statistically accounted for in the final analysis.

Students were informed about the study, and those interested in participating provided written assent in the presence of their teachers and school administrators. Prior to final recruitment, meetings were held with parents of students who assented and with school officials. During these sessions parents and students were informed about the study’s purpose, eligibility criteria, procedures, and equipment. Final enrollment occurred after written parental consent was received. All communications were conducted in English or Igbo, based on participants’ preference.

### 2.3. Sample size

The sample size for the NARN’ia study was informed by similar studies using doubly labeled water (DLW) and accelerometry to assess energy expenditure and physical activity in adolescents, particularly in low- and middle-income countries [55,68–70]. Studies in comparable populations have used sample sizes ranging from 21 to 60 participants. Given the complexity and high cost of DLW analysis and the logistical challenges of conducting field-based research in rural Nigeria, we selected a target recruitment sample size of n = 70 as both practical and methodologically robust. Power analyses conducted using G*Power software (version 3.1) indicate that a sample size of 70 achieves approximately 70–91% power to detect medium effects in multiple linear regression models at a significance level of α = 0.05 when testing between 2–4 predictors [71]. This sample size is expected to be sufficient to detect meaningful associations between total energy expenditure and key variables such as body composition, physical activity, dietary intake, and age etc., while balancing the practical constraints of cost and logistics of conducting research in rural Nigeria with scientific rigor.

### 2.4. Data and sample collection

#### 2.4.1. Total energy expenditure.

**Fig 1:**
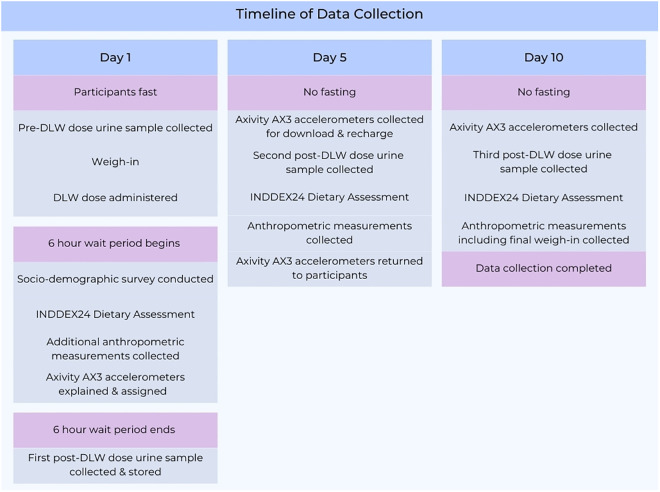
Timeline of Data Collection. DLW is administered and urine samples are collected 0ver a 10-day period. Additionally, socio-demographic, dietary, anthropometric, and activity data are collected.

Data and sample collection will take place three times over a ten-day period between March 7 and May 13, 2024 (Fig 1). On the morning of days 1, 5, and 10, participants will be transported to the Nutrition Assessment Laboratory at the Department of Human Nutrition and Dietetics, Michael Okpara University of Agriculture, Umudike (MOUAU) where data collection will take place. On Day 1, participants will be required to fast beforehand although they will be allowed to drink water.

First, participants will provide ~5 ml of pre-dose urine which will be used to determine each of their isotopic baselines. The time of this and all subsequent urine sample collections will be recorded accurately to the nearest minute. Next, participants will be weighed using an Omron Body Composition Monitor and Scale (BF-511) to the nearest 0.1%. This is a bioelectrical impedance scale that estimates the percentage of body fat, muscle mass, and other tissues in the body by sending a low-level electrical current through the body and measuring the resistance (impedance) to the current flow. Different tissues in the body, conduct electricity differently for example, muscle tissue, which contains more water, conducts electricity better than fat tissue. By analyzing the impedance, the scale can estimate the proportion of different tissues within the body [72]. A dose of DLW (6% H_2_-^18^O, 10% ^2^ H_2_-O) tailored to their body mass at 1 ml per/ kg will be administered to each participant also at a recorded time. While waiting for the DLW to equilibrate in the body, participants will be provided with food and water, and the rest of the data collection will continue. Approximately 6 hours after DLW dose administration, participants will provide another urine sample. On days 5 and 10, participants will not be required to fast before urine sample collection. Samples will be stored and shipped cold (2–8 °C) to the Pontzer Lab at Duke University, USA for isotope enrichment analysis via laser-absorption mass spectrometry. Depletion rates of ^2^H and ^18^O will be used to calculate each participant’s rate of CO_2_ production over the 10-day study period.

#### 2.4.2. Socio-demographic characteristics.

During the waiting period between DLW dosing and the first post-dose urine collection, participants will be asked to fill out a socio-demographic characteristics survey adapted [73]. Trained research assistants will assist participants in filling out the survey with information about their age, household size, parents’ education, occupation, and income.

#### 2.4.3. Dietary assessment.

A 24-hour dietary recall will be conducted electronically using the International Dietary Data Expansion (INDDEX24) Dietary Assessment mobile application on the days of data collection, utilizing the multiple pass method [18]. During the first pass, participants will be asked to recall all the foods and beverages they consumed in general terms, using local meal/recipe names and brand names. In subsequent passes, more detailed information about each food item will be recorded, including the time and location of consumption, ingredients, methods of preparation, portion sizes, and other food-specific details.

#### 2.4.4. Anthropometric measurements of body composition.

In addition to body mass (BM), additional anthropometric measurements such as height, waist, and hip circumference data, and SFT will be recorded using a height ruler, tape measures, and calipers, respectively. On day 10, after the final urine sample is collected, participants will be weighed again using the bioelectrical impedance scale.

#### 2.4.5. Physical activity level.

Daily physical activity will be tracked using the wrist-worn Axivity AX3 triaxial digital accelerometers during the DLW period. Participants will receive the accelerometers on day 1, along with instructions on proper wear and maintenance. They will be instructed to wear the accelerometer at all times on their non-dominant wrist to minimize noise, and to only take it off for water-based activities. Parents and school staff will be asked to help ensure compliance. The accelerometers will be collected on day 10, and the data will be downloaded for analysis. Additionally, participants will be given a physical activity diary to record wear and non-wear times.

## 3. Data analyses

### 3.1. Dietary assessment

The data collected using the INDDEX 24 App will be analyzed on the INDDEX Dietary Assessment Platform using the Analytical Reports features which provide simple summary statistics of the dietary survey data. The results will be compared against standards of recommended daily energy and nutrient requirements for adolescents [74] to determine the adequacy of energy and nutrient intake.

### 3.2. Accelerometry

Activity log data from the accelerometers will be downloaded as.csv files using the AX3 software and imported into R software [75] for raw accelerometer data processing using the GGIR v.3.1−1 [76,77] package. Analysis will include the quantification of time of non-wear which will be validated against each participant’s physical activity diaries and to estimate times of physical activity, inactivity, and sleep.

### 3.3. Anthropometric Data Analyses

Participants’ body mass index (BMI) will be calculated using WHO (World Health Organization) Anthroplus software and assessed against the WHO BMI-for-age (5–19 years) girls z-score charts into categories of thin (<−1SD), healthy (0), overweight (>+1SD), and obese (>+2SD) [78,79]. Additionally, other anthropometric ratios, such as waist-to-hip ratios, will be calculated and categorized according to the WHO criteria [80]. Waist-to-hip ratios less than 0.85 will be considered healthy, while ratios above 0.85 will be classified as indicating a higher health risk.

#### 3.3.1. Skinfold thickness.

SFT will be measured at the biceps, triceps, subscapular, and suprailiac skinfolds according to the International Society for the Advancement of Kinanthropometry (ISAK) guidelines [37]. The values will be used to estimate each participant’s FM and FFM using adapted equations [2, 81, 82]. SFT is being collected to be compared against deuterium dilution for validation in this population, so that we can use skinfolds and/or bioimpedance alone in future work.

#### 3.3.2. Total body water.

TBW will be estimated using deuterium dilution [40], using a similar approach and notation related to that used by the International Dietary Energy Consultancy Group (IDECG). Assessment of TBW is integrated into the DLW technique as an intermediary step for calculating carbon dioxide production [40,43].

#### 3.3.2. Fat free mass and fat mass.

The measured BM and calculated TBW will be used to calculate FFM and FM. FFM will be derived from TBW using a hydration coefficient (0.732) [40] i.e., the fraction of FFM comprised of water [40,83,84]. FM will be derived by subtracting FFM from BM and its percentage will also be derived [40]:


$$\text{FFM }(\text{kg})\;=\;\frac{\text{TBW}}{\text{hydration coefficent}}$$ (1)

Thus,


FM (kg) = BM−FFM (2)

$$∴\text{ FM }(\%)\;=\;\frac{\text{FM}}{\text{BW}}\text{ x 100}$$ (3)

The participants FM percentages will be categorized into 4 categories: under fat, normal fat, over fat, and obese using reference curves [85].

## 4. Discussion

Adolescence marks a crucial phase characterized by rapid physical growth, maturation, and dynamic changes in body composition. This developmental phase, bridging childhood and adulthood, is pivotal for establishing lifelong health trajectories. The prevalence of malnutrition among adolescent girls in Nigeria underscores the urgent need for targeted nutritional strategies that meet their developmental needs. NARN’ia employs a multifaceted approach to comprehensively evaluate energy expenditure, physical activity levels, and body composition to provide the holistic understanding that is needed to inform nutritional interventions. The use of DLW method represents a significant advancement in measuring energy expenditure in this region, contributing novel insights into their energy metabolism and nutritional requirements. Furthermore, the integration of accelerometry for assessing physical activity will address the challenge of quantifying daily activity patterns in this population to offer a more robust measure of TEE.

The insights generated from NARN’ia have significant implications for policy makers and stakeholders involved in adolescent health and nutrition. By outlining precise energy requirements and nutritional patterns among adolescent girls in Nigeria, our findings will inform evidence-based policies aimed at addressing nutritional deficiencies. In summary, this study represents a pioneering effort to comprehensively assess energy requirements among adolescent girls in Nigeria. By addressing gaps in current knowledge and methodologies, our research will contribute valuable data to global efforts aimed at improving nutritional interventions and promoting healthy development during adolescence and guiding future research focused on improving the nutritional status and overall well-being of adolescent girls worldwide.

## 5. Trial status

Data collection for Phase 1 of this study was completed in May 2024, and preliminary data analysis is currently underway. Recruitment for Phase 2 has been completed, and data collection is actively ongoing. The study remains on track for final analysis and dissemination following the completion of both phases.

## Abbreviations

BM: Body mass
BMI: Body mass index
DLW: Doubly labeled water
FM: Fat mass
FFM: Fat free mass
HIC: High income countries
IDECG: International Dietary Energy Consultancy Group
INDDEX: International Dietary Data Expansion
ISAK: International Society for the Advancement of Kinanthropometry
LMIC: Low and middle income countries
MOUAU: Michael Okpara University of Agriculture, Umudike
SFT: Skinfold thickness
TBW: Total body water
TEE: Total energy expenditure
WHO: World Health Organization
